# Investigation into In Vitro and In Vivo *Caenorhabditis elegans* Models to Select Cheese Yeasts as Probiotic Candidates for their Preventive Effects against *Salmonella* Typhimurium

**DOI:** 10.3390/microorganisms8060922

**Published:** 2020-06-18

**Authors:** Philippe Veisseire, Muriel Bonnet, Taous Saraoui, Cyril Poupet, Olivier Camarès, Marylise Gachinat, Cécile Callon, Guy Febvre, Christophe Chassard, Stéphanie Bornes

**Affiliations:** 1 Université Clermont Auvergne, INRAE, VetAgro Sup, F-15000 Aurillac, France; muriel.bonnet@uca.fr (M.B.); taous.saraoui@uca.fr (T.S.); cyril.poupet@uca.fr (C.P.); olivier.camares@uca.fr (O.C.); marylise.paquet@uca.fr (M.G.); cecile.callon@inrae.fr (C.C.); christophe.chassard@inrae.fr (C.C.); stephanie.bornes@uca.fr (S.B.); 2 Université Clermont Auvergne, Laboratoire Météorologie Physique, CNRS, F-15000 Aurillac, France; guy.febvre@uca.fr

**Keywords:** Probiotics, cheese yeasts, *Salmonella* Typhimurium, *Caenorhabditis elegans*

## Abstract

The design of multiscale strategies integrating in vitro and in vivo models is necessary for the selection of new probiotics. In this regard, we developed a screening assay based on the investigation of the potential of yeasts from cheese as probiotics against the pathogen *Salmonella* Typhimurium UPsm1 (ST). Two yeasts isolated from raw-milk cheese (*Saccharomyces cerevisiae* 16, Sc16; *Debaryomyces hansenii* 25, Dh25), as well as *S. cerevisiae* subspecies *boulardii* (CNCM I-1079, Sb1079), were tested against ST by applying in vitro and in vivo tests. Adherence measurements to Caco-2 and HT29-MTX intestinal cells indicated that the two tested cheese yeasts presented a better adhesion than the probiotic Sb1079 as the control strain. Further, the Dh25 was the cheese yeast most likely to survive in the gastrointestinal tract. What is more, the modulation of the TransEpithelial Electrical Resistance (TEER) of differentiated Caco-2 cell monolayers showed the ability of Dh25 to delay the deleterious effects of ST. The influence of microorganisms on the in vivo model *Caenorhabditis elegans* was evaluated by measuring the longevity of the worm. This in vivo approach revealed that this yeast increased the worm’s lifespan and protected it against ST infection, confirming that this in vivo model can be useful for screening probiotic cheese yeasts.

## 1. Introduction

The gut microbiota is dominated by bacteria, but eukaryotic microorganisms also contribute to the human microbiome [1,2]. This complex community interacts with the host, greatly impacts human health and physiology, and is essential for gastrointestinal homeostasis [3]. Perturbations in the gut microbiota composition may represent an important risk factor for disease [4]. Food, and particularly cheese, obtained by the activity of bacteria and yeasts, should be a source of eukaryotic microorganisms, some of which may have a probiotic potential. The Food and Agriculture Organization of the United Nations (FAO) and the World Health Organization (WHO) [5] defined probiotics as live microorganisms that, when administered in adequate amounts, confer a health benefit on the host. The majority of the probiotic strains that have been studied are lactic bacteria. However, more recently, research into yeasts with potentially beneficial influences on human health has mainly revolved around *Saccharomyces cerevisiae* subspecies *boulardii* CNCM I-1079. This is one of the most well-known probiotic yeasts for human use and available on the market [6] and has been included in numerous controlled trials. Yeasts are not affected by antibacterial treatments, and this is one of the reasons why *S. boulardii* is prescribed for the prevention of antibiotic-associated diarrhea [7]. This strain has shown a beneficial effect on inflammatory bowel diseases such as Crohn’s disease [8] by impacting the inflammatory cytokine production of the intestinal epithelial cells [9]. Little is known about the interaction between non-*Saccharomyces* yeasts and the human microbiota, but other yeast species have been reported to have potential probiotic properties, including *Debaryomyces hansenii*, *Kluyveromyces lactis*, *Yarrrowia lipolytica*, and *Issatchenkia occidentalis* [10,11,12]. These species are frequently associated with dairy products such as raw-milk cheeses [13,14]. Furthermore, these species have the Qualified Presumption of Safety (QPS) status in the European Union and are commonly found and used in food fermentation [15]. These facts could support and help the applications for human use.

Different approaches for new probiotic screening can highlight different physiological characteristics and mechanisms of action. Indeed, potentially probiotic microorganisms generally need to be able to survive in the gastrointestinal tract in order to function in the gut environment. They must withstand acid gastric juice, bile salts, low pH, and internal human body temperature. Moreover, it is preferable that they adhere to the epithelial cells in order to better exert their effect against intestinal pathogens. They can function as a microbial barrier by preventing pathogen binding [16] and modulation of the immune system [17,18], as well as by the production of inhibitory compounds, such as organic acid, oxygen catabolites, and proteinaceous compounds [19]. Adhesion ability is strain-dependent, and an evaluation of this characteristic is recommended for the selection of a probiotic [19,20]. Recent research has shown the need to combine in vitro and in vivo approaches with animal models as a preface to human studies in order to decipher probiotic functions and mechanisms of action [21]. However, in vivo assays are expensive and time-consuming as they require a large number of animals with the ensuing ethical problems. This would explain why, recently, there has been a noticeable increase in the use of the soil nematode *Caenorhabditis elegans* in microbiology studies. Another advantage of this model is that its intestinal cells are structurally similar to those in humans [22], and its body is transparent [23]. This worm has already been used successfully as a model organism for the simple, rapid and reliable screening of potential probiotic bacteria [23] and, more especially, to evaluate the protective effect of *Lactobacillus spp.* against *S.* Typhimurium [24]. *C. elegans* has also emerged as a valuable model host in which to study the pathogenicity of yeasts [25,26,27,28] but has rarely been used to evaluate the possible probiotic activities of yeasts [29]. Recently, Kunyeit et al. [12] chose this model to highlight the protective effect of yeasts against pathogenic non-*albicans Candida* species. *C. elegans* has never been before considered for the evaluation of the preventive effects of yeasts against pathogenic bacteria.

*Salmonella enterica* serovar Typhimurium is a major cause of infectious disease in animals (i.e., poultry, pig, human). Human contamination can occur via goat milk and cheese consumption [30], as a substantial percentage of animals carry this microorganism in their rumen and feces [31]. The pathogenesis of *Salmonella* requires, in particular, the adhesion to and the invasion of the intestinal epithelial cells [32]. This bacterium then induces the disruption of the host cell tight junctions, leading to a decrease in the TransEpithelial Electrical Resistance (TEER) [33]. Indeed, TEER is a widely accepted quantitative technique to measure the integrity of tight junctions. TEER values are strong indicators of the integrity of cellular barriers. They can be performed in real time without cell damage and generally are based on measuring ohmic resistance. To investigate *Salmonella* adhesion and invasion, Gagnon et al. [34] used human colonic Caco-2 and mucus-secreting HT29-MTX cell lines. The results showed the influence of *Salmonella* serovar on cell adhesion as well as the positive effects of mucus on the infection. These in vitro cell models have also been widely selected for in vitro attachment and mechanistic studies [35]. This pathogen can survive for a long time in stored cheese, and one approach for inhibiting it is the use of lactic acid bacteria. Yeasts can also be considered for this purpose.

In this study, two yeasts isolated from raw-milk cheese (*Saccharomyces cerevisiae* 16, Sc16; *Debaryomyces hansenii* 25, Dh25), as well as *Saccharomyces cerevisiae* subspecies *boulardii* (CNCM I-1079, Sb1079) were experimented against *Salmonella* Typhimurium (UPsm1 strain, ST) by applying in vitro recognized tests (human cells) and an innovative *C. elegans* in vivo test. Tolerance to low pH and bile was evaluated, and the ability of the yeasts to adhere to human epithelial intestinal cells was observed using the Caco-2 and HT29-MTX cell lines. We also assessed the impact of the yeasts on the TEER of the differentiated monolayers of the Caco-2 cells, and the ability of Dh25 to delay the deleterious effects of ST on the integrity of the monolayers cells. An in vivo approach using the nematode *C. elegans* was performed by testing its ability to survive and to extend its lifespan in the presence of the yeasts and/or the pathogen and, thus, to consider its possible use for the screening of potential probiotic yeasts.

## 2. Materials and Methods

### 2.1. Yeasts, Bacteria Strains, and Growth Conditions

All of the strains—*Salmonella enterica* serovar Typhimurium UPsm1 (ST)*, Saccharomyces cerevisiae* (Sc16 and Sc52 strains)*, Debaryomyces hansenii* (Dh14 and Dh25 strains), *Yarrowia lypolitica* (3CP5 and 4PO1 strains) and *Kluyveromyces lactis* (Kl1 and Kl6 strains), isolated from cheese—came from the UMRF (Unité Mixte de Recherche sur le Fromage) collection. *Saccharomyces cerevisiae* subspecies *boulardii* CNCM I-1079 (*S. boulardii*, Sb1079) was obtained from Ultralevure (Lallemand, Toulouse, France). *Escherichia coli* OP50 (standard food for nematode) was obtained from the *Caenorhabditis* Genetics Center (CGC, Minneapolis, USA). All strains were stored at −80 °C in their culture media with 20% glycerol (Biosolve, Dieuze, France). For all experiments, *S.* Typhimurium was aerobically grown at 37 °C for 24 h in 50 mL Brain–Heart Infusion (BHI) broth (Biokar Diagnostics, Pantin, France), and yeasts were aerobically grown at 25 °C for 48 h, in 50 mL Yeast Extract–Peptone–Glucose (YPG) broth, at pH = 5.2 (40 g/L glucose (Sigma-Aldrich, St-Louis, USA), 5 g/L yeast extract (Biokar Diagnostics) and 5 g/L peptone (CONDA pronadisa, Madrid, Spain)). *E. coli* OP50 was aerobically grown at 37 °C for 24 h, in 50 mL Lysogeny Broth (LB) (Biokar Diagnostics).

### 2.2. Human Cell Cultures

The human colon carcinoma cell line, Caco-2 [36] and the mucus-secreting HT29-MTX cell line, provided by Dr. Thécla Lesuffleur (INSERM UMR S 938 Paris, France), were grown in Dulbecco’s Modified Eagle’s Minimal essential medium (DMEM) (Life Technologies, Villebon sur Yvette, France) supplemented with 20% or 10%, respectively, of heat-inactivated fetal calf serum (Life Technologies). The cells were maintained at 37 °C with 5% CO_2_ in air atmosphere.

### 2.3. Determination of Acid pH and Bile Tolerance of Yeasts

As described previously [37], acid tolerance at pH 2.5 was determined in gastric juice (Bacto Peptone 30 g/L (FISHER Bioblock Scientific, Illkirch, France), sodium chloride 99.5% 15 g/L (ACROS ORGANICS, Geel, Belgium), Pepsin A 3.2 g/L (SIGMA, Saint Quentin-Fallavier, France)) adjusted with HCl 0.1 M (DUTSCHER, Brumath, France). A suspension of the yeast strains from a 20 h culture, corresponding to approximately 10^7^ colony-forming units (CFU)/mL, was added to 10 mL of the gastric juice solution. Viable cells were measured after 0, 45, and 90 min by plating serial dilutions of aliquots onto Oxytetracycline Glucose Agar (OGA) base (Biokar, France), supplemented with 0.1% (w/v) oxytetracycline. The Petri dishes were incubated for 3 days at 25 °C.

Bile tolerance was determined in 20 mL of YPG broth with or without 3% of bile salts (SIGMA). The number of viable cells was determined after 0, 60, 120, 180, and 240 min of incubation by plating the serial dilutions of aliquots onto OGA Petri dishes incubated for 3 days at 25 °C. All assays were performed in triplicate.

### 2.4. In Vitro Yeasts Adhesion Assay

The adhesion capacities of the yeast strains were examined using the Caco-2 and the HT29-MTX cell lines. Human cells were seeded at a concentration of 3.5 × 10^5^ cells per well in 24-well plates (Dutscher, Brumath, France) and placed in growth conditions overnight and for 15 days.

Overnight cultures of yeast strains were harvested by centrifugation (1500g for 15 min), washed once with phosphate-buffered saline (PBS), and suspended in 9 mL of DMEM supplemented with 20% (Caco-2) or 10% (HT29-MTX) fetal calf serum. The final multiplicities of infection (MOIs, ratios between yeasts and human cells) tested were 0.1, 1, 10, and 100. The plates were incubated for 3 h at 37 °C under 5% CO_2_. After incubation, the wells were washed three times with 1 mL of PBS, and the cell monolayer with adhering yeasts was detached by the addition of 1 mL of trypsin solution (Life Technologies) and resuspended in sterile water. The number of viable yeasts was determined by plating serial dilutions of the suspensions onto YPG agar plates after 48 h at 25 °C. Each adhesion assay was performed in triplicate.

### 2.5. Growth Inhibition of *Salmonella* Typhimurium by Yeasts

For the ST and yeast growth monitoring in pure culture and co-cultures, cells were harvested at 1500g for 15 min. Microbial cell pellets were resuspended in 2 mL of their respective medium culture, and cell concentrations were then determined using a Petroff–Hausser Counting Chamber. Mixtures of 5 mL of BHI and 5 mL of YPG were then co-inoculated with ST or yeasts, respectively, to finally obtain 10^6^ cells per mL of each microorganism in 50 mL conical bottom tubes. Pure culture controls were made in the same conditions. They were then aerobically incubated (250 rpm) for 0 h, 24 h, and 48 h at 37 °C. After these incubations, serial dilutions were performed and 100 µL were plated onto BHI and YPG agar plates for ST and yeast numerations, respectively. The number of viable pathogens and yeasts was determined by counting the colony-forming units (CFU) after an overnight incubation at 37 °C for the ST and a 48 h incubation at 25 °C for the yeasts. Each assay was performed in triplicate with at least three independent cultures.

### 2.6. Caco-2 TransEpithelial Electrical Resistance (TEER) Assay

Caco-2 cells (400 µL at a density of 3.5 × 10^5^ cells/mL) were seeded on Merck-Millipore^®^ Transwell^®^ polycarbonate cell culture inserts with a 0.4 µm filter membrane and a 0.6 cm^2^ surface area. They were placed in Cellstar^®^ 24-well plates. The values of TEER were determined by measuring the potential difference between the two sides of the cell monolayer using a Millicell^®^ ERS-2 m (Millipore^®^, Billerica, MA, USA) connected to a pair of chopstick electrodes. TEER measurements and medium changes were carried out every two days to maintain optimal culture conditions and to make sure of the complete maturity of the cell monolayer i.e., 6 days of culture in our experimental conditions.

To evaluate the influence of yeasts and ST on the epithelial cell barrier, mature cell cultures were washed with PBS prior to the addition of 400 µL of each microorganism, which was resuspended in DMEM at a final concentration of 10^6^ CFU/mL. TEER measurements were made every hour for 8 h and then once again at 24 h. 

In order to evaluate the influence of Dh25 on the TEER modulation by ST, co-cultures of 24 h, 48 h, 72 h, and 96 h were made and resuspended in DMEM. A volume of 400 µL of these four co-cultures was inoculated into the monolayer cells. TEER measurements were made every hour for 16 h, and once at 24 h, in comparison with 400 µL of ST alone, at a concentration of 6.3 log CFU/mL.

The resistance value of each individual Transwell was calculated by subtracting the resistance of a coated Transwell without cells from the total resistance (cell epithelium with or without microorganism plus filter insert). Values at 0 h were normalized to 100%. Data were presented as the average of three independent experiments.

### 2.7. *C. elegans* Maintenance and Synchronization

*C. elegans* Bristol strain N2 (wild-type) and the mutant AU37 (*glp-4*(*bn2*) I; *sek-1*(*km4*) X) with enhanced sensitivity to pathogens were obtained from the *Caenorhabditis* Genetics Center (CGC). The worms were maintained at 20 °C and 15 °C, respectively, on Nematode Growth Medium agar containing 4 g/L Yeast extract (NGMY), seeded at least 24 h at 37 °C with *E. coli* OP50 [38]. For synchronization, eggs and gravid worms were washed with M9 buffer and centrifuged at 190g for 2 min. The pellet was resuspended in 5 mL of Worm Bleach (2.5 mL, 1.5 pure sodium hypochlorite and 1 mL sodium hydroxide 5M), mixed vigorously for 3 min until adult worm body disruption. Sodium hypochlorite action was then immediately stopped by using 10 mL of M9 buffer and afterward centrifuged at 190 *g* for 2 min. The pellet containing the eggs was washed twice with 10 mL of M9 buffer and then, finally, resuspended in 20 mL of M9 buffer. The eggs were incubated on a shaker (150 rpm) at 25 °C for 24 h to obtain L1 larvae. These larvae were settled by centrifugation at 190 *g* for 2 min and were finally resuspended in 1 mL of the remaining supernatant. One hundred microliters were then transferred onto NGMY plates, seeded with *E. coli* OP50, and incubated until the worms reached the L4/young adult stage.

### 2.8. *C. elegans* Longevity Assay

In a solid medium (Petri dishes of 5 cm diameter), to evaluate the effect of yeasts and *S.* Typhimurium on the host (*C. elegans* N2), the lifespans of the nematode were measured according to the method of Lee et al. [24] and Poupet et al. [39]. At the L4/young adult stage, the worms were divided into several groups. The control group was fed ad libitum on *E. coli* OP50, and the experimental groups on one microbial strain. The medium used was NGMY supplemented with 0.12 mM 5-fluorodeoxyuridine (FUdR, Sigma, Saint-Louis, USA) to avoid egg hatching. Worms were kept at 20 °C. The number of living nematodes was scored every day until the death of the last worm. A worm was considered dead when it failed to respond to gentle touch. This assay was performed in three independent experiments with three Petri dishes per condition.

### 2.9. *C. elegans* Survival Assay

For the liquid medium condition, nematodes (L4/young adult stage N2 strain) were washed from *E. coli* OP50-seeded plates with M9 buffer and placed on NGMY + 0.12 mM FUdR plates containing Dh25, Sc16 or Sb1079 for 24 h. The medium used for *S.* Typhimurium was BHI. The worms were then washed twice with M9 medium and transferred into the wells of a 6-well microtiter plate (approximately 50 worms per well). Each well contained 2 mL of liquid assay medium (20% BHI and 80% M9) + 0.12 mM FUdR. The plates were incubated at 20 °C. The nematodes were observed daily until the death of the last worm. They were considered dead when they did not move after mechanical stimulation on the dish. This assay was performed in three independent experiments with n = 3 wells per group.

### 2.10. Effects of *D. hansenii* on *S.* Typhimurium Infection in the *C. elegans* Survival Assay

To evaluate the preventive effects of *D. hansenii* against *S.* Typhimurium, AU37 nematode survival was measured in a liquid medium according to the work of de Barros et al. [40] and Poupet et al. [39] with some modifications. A synchronous population of AU37 worms (stage L1) was cultured on NGMY plates, with a 24 h culture of *E. coli* OP50, at 25 °C for 48 h. For the preventive tests, the nematodes were washed with M9 buffer and placed on NGMY plates containing Dh25 or *E. coli* OP50 for 24 h. They were then washed twice with M9 buffer and transferred onto BHI agar containing ST for 4 h. Infected worms were washed twice with M9 medium and transferred into the wells of a Corning^®^ 6-well microtiter plate (50 worms per well). Each well contained 2 mL of liquid assay medium (20% BHI and 80% M9). For the non-preventive treatment groups, the worms were placed on Dh25 and *E. coli* OP50 for 24 h or for 4 h with ST. They were then washed and transferred onto microtiter plates and incubated at 25 °C. The nematodes were observed until the death of the last worm. They were considered dead when they did not move after mechanical stimulation on the dish. This assay was performed in three independent experiments with n = 3 wells per group.

### 2.11. Statistical Analysis

The *C. elegans* survival assay was examined by using the Kaplan–Meier method, and differences were determined by using the log-rank test with R software version 3.5.0 [41] survival [42] and survminer [43] R packages. Each experiment was performed in three independent experiments with n = 3 wells per group. The null hypothesis H0 is that of the equality of the survival functions between two groups. A *p*-value ≤ 0.05 was considered as significant. Other statistical analyses were performed using R software version 3.5.0 [40] with a single-factor analysis of variance (ANOVA) followed by Fisher’s Least Significant Difference (LSD) test.

## 3. Results and discussion

### 3.1. Acidic pH and Bile Tolerance

The survival of probiotics during gastric transit is important for the colonization of the gastrointestinal tract. The ability to survive at pH 2.5 for 45 and 90 min, or a bile concentration of 3%, was the first step to determine the probiotic potential of the selected isolates of the following species: *D. hansenii*, *S. cerevisiae*, *Y. lipolytica*, and *K. lactis* from cheese. All the yeast strains tested were able to grow under conditions simulating the passage through the gastrointestinal tract (Table S1). Based on their capabilities to resist both acid and bile, Dh25 and Sc16 were chosen as probiotic candidates to be tested on our in vivo model. The well-known probiotic Sb1079 was included as the control strain [44,45,46,47]. Usually, the acid tolerance test is performed at pH 3, but a pH value of 2.5 is more selective [48]. At this pH, the highest percentage of survival was observed (Table 1a) for Sb1079 (91.1 ± 9.0% after 90 min). Dh25 also presented a high resistance with 85.5 ± 3.0% survival. In the same conditions, Sc16 presented a 50% decrease in viability between 45 and 90 min, to finally reach 54.2 ± 2.7%.

Bile is an amphiphilic substance secreted into the small intestine. It can damage the membranes of microorganisms [49]. The percentage of survival of all the strains tested (Table 1b) decreased in relation to the length of contact time with the 3% bile condition (from 1 to 4 h). Sb1079 and Dh25 showed high resistance to bile, with a 73.5 ± 4.9% and 68.9 ± 16.1% survival rate, respectively, at 4 h. In comparison, Sc16 was the most sensitive strain, with 39.2 ± 6.7% survival.

In general, yeasts associated with food show a high survival rate in gastrointestinal tract conditions [50,51]. In our study, Dh25 was the most suitable yeast strain for gut colonization. However, we can hypothesize that a sufficient number of viable cells of the other two species could also reach the small intestine and, once there, exhibit their functional activity.

### 3.2. In Vitro Adhesion Assay 

The adhesion of probiotics to the epithelial intestinal cells is also an important prerequisite for mucosal colonization. It is an effective way to exclude pathogens. The adhesive capacity of the selected yeasts was examined in vitro with the Caco-2 and HT29-MTX cell lines. Four multiplicities of infection (MOIs) were tested (100, 10, 1, and 0.1). The more the MOI decreased, the more the adhesion capacities increased (Table 2). Indeed, the best adhesion percentage was obtained at an MOI of 0.1 for both of the cell types. In addition, when the concentration of yeasts was high (high MOIs), the lower adhesion may be due to yeast auto-aggregation reducing the amount of available space on the cell it has adhered to [52]. The lowest adhesion capacity at the MOI of 0.1 was observed with Sb1079 (3.60 ± 0.028% with Caco-2 cell line and 1.15 ± 0.270% with HT29-MTX). With Dh25, the adhesion to the Caco-2 and to the HT29-MTX cells reached 4.12 ± 1.701% and 1.56 ± 0.010%, respectively. The adhesion ability of Sc16 was the highest in the Caco-2 (7.26 ± 0.088%) compared with the HT29-MTX (2.61 ± 0.24%) cells. Although the three tested yeasts tended to follow the same trend in both of the intestinal cell lines, they exhibited a lower percentage of adhesion to the mucus-producing cell line HT29-MTX. These results are in agreement with Zivkovic et al. [10], who mentioned that, generally, yeasts isolated from traditional cheeses manufactured in Serbia and Croatia show better adhesion to the Caco-2 cell line than to the HT29-MTX. They suggested that the presence of mucin hinders the availability of the cells as receptors for yeasts. Ochangco et al. [11] also showed that Dh25 adhered better to Caco-2 cells, which was explained by the fact that these cells had a smooth cell surface, creating easier access for the yeasts [53]. The two tested cheese yeasts presented a better adhesion capacity than the control yeast, Sb1079. These results show that it is of interest to screen these microorganism strains for their probiotic properties, especially Dh25, which was found to survive better under anaerobic conditions than under aerobic conditions, as does *S. boulardii* [11]. Indeed, the conditions become increasingly anaerobic during the passage through the gastrointestinal tract [54].

### 3.3. Growth Inhibition of *Salmonella* Typhimurium by Yeasts

Whatever the yeast tested, the number of ST was always lower in a pure culture than in a co-culture, whether 24 h or 48 h (Table 3). In a pure culture, with an initial cell concentration of ST of 6.47 log CFU/mL, the bacterial population increased and reached 8.88 log CFU/mL at 24 h and 8.71 log CFU/mL at 48 h. When ST was co-cultured with Sb1079, no significant difference was observed at 24 h. As for the other two cheese yeasts, the ST population was significantly lower than in a pure culture. At 48 h, the three yeasts clearly decreased the ST population by over 2 log CFU/mL. These results showed the inhibitory activity of the yeasts against the ST and were in accordance with Lima et al. [55], who demonstrated that *S. cerevisiae* strains from Brazilian kefir-fermented milk presented an antagonistic activity against Gram-negative bacteria. Similar results were obtained by Roussel et al. [56] with a *S. cerevisiae* strain on an enterotoxigenic *E. coli*. On the contrary, Binetti et al. [57] showed that yeasts from autochthonal cheese starters did not inhibit the growth of *Salmonella* Enteritidis, *E. coli,* or *Staphylococcus aureus*. Perricone et al. [58] also demonstrated that yeasts isolated from Altamura sourdough did not have antagonistic activity against *Listeria monocytogenes*, *Yersinia enterocolitica*, *E. coli* O157:H7, or *S. aureus*. All these results show that, as in the case of bacteria, the antipathogenic properties are species- and strain-dependent in yeasts and that the method used is likely to influence these results.

### 3.4. Evolution of Yeasts and *Salmonella* Typhimurium-Induced Epithelial Caco-2 Cell Barrier Function

**Figure 1:**
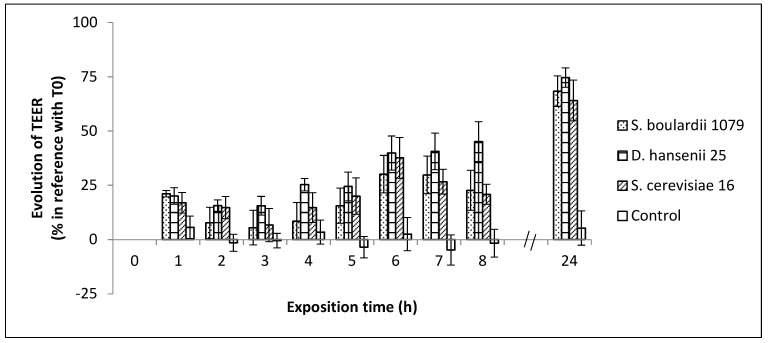
Change in TransEpithelial Electrical Resistance (TEER) level of yeast-exposed Caco-2 cells during apical incubation with *S. boulardii* 1079, *D. hansenii* 25, *S. cerevisiae* 16, and Dulbecco’s Modified Eagle’s Minimal essential medium (DMEM) control medium throughout the 24 h incubation period, expressed by reference to resistance measured at time 0. The values are mean ± SD (error bars) of three independent experiments. A high enhancement of the TEER is observed in the presence of the yeasts.

As a measure of epithelial cell barrier function in vitro, we assessed the TEER across the differentiated cell monolayers of the human colonic cell line Caco-2; an established approach often applied for the evaluation of potential probiotic properties of microorganisms [59]. It is now well established that a multiplicity of factors can affect TEER measurements [60,61]. Indeed, in our experimental conditions, monitoring of intestinal epithelium resistance showed that the TEER values stabilized after 6 days (Figure S1) when the formation of the tight junctions in the Caco-2 cells was optimal. The differentiated monolayers of Caco-2 cells were then exposed to the three strains of yeasts (10^6^ CFU/mL) in order to evaluate the influence of these microorganisms on the integrity of the epithelial cell barrier. Caco-2 cells were used instead of HT29-MTX because yeasts have greater adhesion properties on the first cell line, which could promote the anti-*Salmonella* action. Using *S. boulardii* as a control, we wanted to determine if Dh25 and Sc16 were good candidates to promote cell barrier integrity. Figure 1 shows that after only one hour of cell exposition, the transepithelial electrical resistance increased significantly, whatever the tested yeast, when compared to the control cells. The TEER values continued to increase progressively until the end of the experiment (24 h). It clearly appears that the three tested yeasts significantly enhanced transepithelial resistance. These results are in accordance with similar studies using non-*Saccharomyces* yeasts [50,62].

Taking into account the above findings, the Dh25 was considered as the best candidate among the tested cheese yeasts and was thus selected to study its antimicrobial properties. To determine if Dh25 had an influence on cell barrier function and/or on the virulence of ST, we exposed the Caco-2 cell monolayers to ST on its own, and to 24, 48, 72, and 96 h co-cultures (Table 4). After 24 h of co-culture, ST and Dh25 concentrations increased from 6.40 ± 0.10 and 5.93 ± 0.21 log CFU/mL to 8.08 ± 0.58 and 6.60 ± 0.35 log CFU/mL, respectively. From 48 h to 96 h of co-culture, the population of viable ST and Dh25 decreased progressively to finally reach 6.79 ± 0.09 and 4.15 ± 0.85 log CFU/mL, respectively.

**Figure 2:**
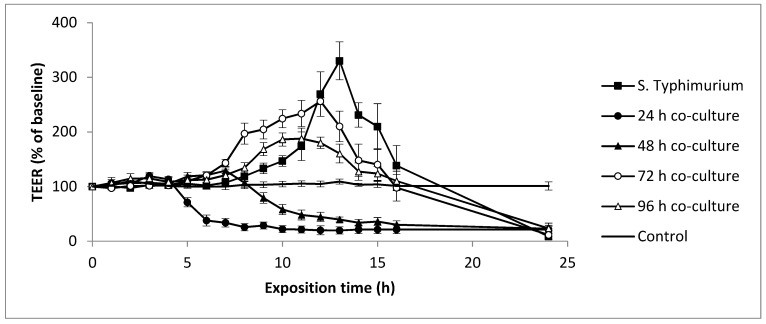
*S*. Typhimurium culture and *D. hansenii* 25/*S.* Typhimurium co-culture (24, 48, 72, and 96 h) modulations of human epithelial cell barrier function. Control with DMEM medium only. The values are mean ± SD (error bars) of three independent experiments. An increased co-culture duration delayed the deleterious effect of *Salmonella* on the TEER.

When 6.3 log CFU/mL of ST alone were applied to the Caco-2 cell barrier (Figure 2), no change in TEER values was observed during the first six hours of the exposition. Values then began to progressively increase up to 330% of the initial value after 13 h of contact. After this period, TEER values rapidly decreased, reaching 138% at 16 h, and finally 8% after 24 h of exposition. These results are in accordance with those of Smith et al. [62]. *S.* Typhimurium is an intracellular bacterial pathogen that resides and proliferates within a membrane-bound vacuole in the epithelial cells of the gut, or freely within the cell cytosol, depending on the host cells [63]. Cellular invasion and intracellular survival are critical virulence stages for this pathogen. It is possible that the increase in the volume of the Caco-2 cells, induced by the proliferation of the ST, enhanced the TEER values until 13 h. It is then essential for the ST to exit the infected cell for its dissemination and transmission to other hosts. These cells are extruded from the monolayer and break, explaining the strong decrease in TEER observed from 13 h of contact with the pathogen to the end of the experiment. It would be interesting to use scanning electron microscopy during TEER experiments to verify this hypothesis.

When Caco-2 cells were exposed to 24 h of a Dh25/ST co-culture, right after the 4 h point of exposition, there was a drastic decrease in the TEER values over the next 4 h, finally reaching a constant value, approximately 20% of the initial value, until the end of the experiment. This can be explained by the enhanced virulence of ST, which is correlated with its multiplication, as mentioned above (from 6.40 ± 0.10 log CFU/mL to 8.08 ± 0.58 log CFU/mL, Table 4). When a 48 h co-culture was applied onto cell layers, the TEER values also decreased, albeit progressively, but not until after 8 h. Indeed, the viable population of ST decreased from 8.08 ± 0.58 to 6.90 ± 0.13 log CFU/mL from 24 h to 48 h of co-culture (Table 4), suggesting that, in this condition, Dh25 exerted a negative effect on the viability of ST. The co-cultures of 72 h and 96 h globally showed a similar pattern. The TEER values increased until 12 h and 11 h, respectively, and subsequently decreased to 20% at the 24 h point (Figure 2). In co-cultures of 48 h, 72 h, and 96 h, the ST viable population was constant (Table 4). The delays in the decrease of the TEER values could be explained by the diminution of virulence at 72 h and 96 h, either due to the presence of the Dh25 or to starvation.

### 3.5. Influence of Yeasts and *Salmonella* Typhimurium on the Longevity and Survival of *Caenorhabditis elegans* and the Preventive Effects of *Debaryomyces hansenii* 25

**Figure 3:**
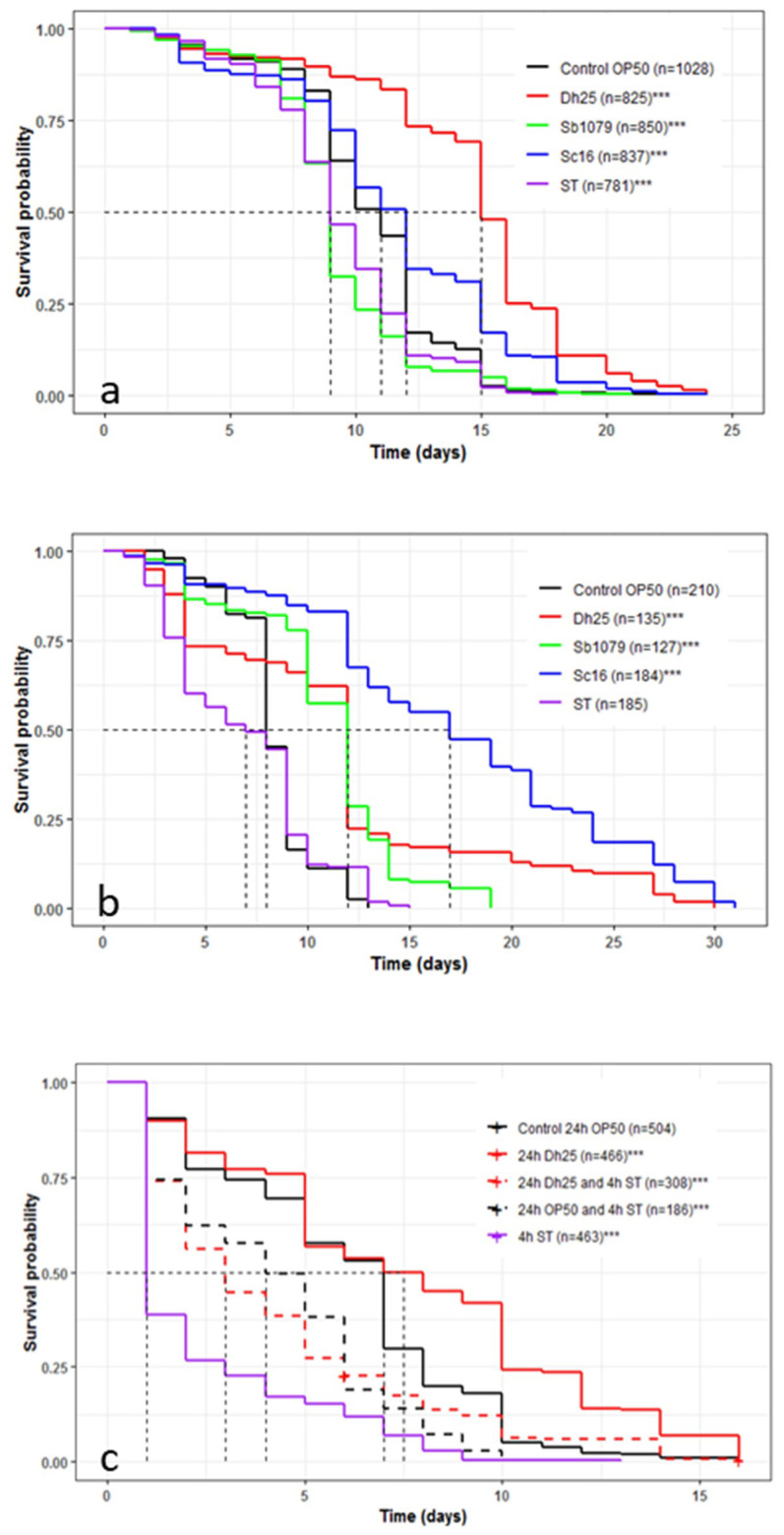
(**a**) Influence of *D. hansenii* 25 (Dh25), *E. coli* OP50, *S. boulardii* 1079 (Sb1079), *S. cerevisiae 1*6 (Sc16), and *S.* Typhimurium (ST) on the longevity of the *C. elegans* wild-type N2 strain in a solid medium. (**b**) Influence of Dh25, *E. coli* OP50, Sb1079, Sc16, and ST on survival of the *C. elegans* wild-type N2 strain in a liquid medium. (**c**) Preventive effects of Dh25 on ST survival of the *C. elegans* mutant AU37 strain in a liquid medium. Mean survival, with half of the population dead, is represented on the abscissa. The asterisks indicate the *p*-values (log-rank test) against *E. coli* OP50 (***, *p* < 0.001). *C. elegans* AU37 mutant is more sensible to the pathogenic bacterium *Salmonella* Typhimurium and is protected by the yeasts *Debaryomyces hansenii* 25.

We observed whether feeding the *C. elegans* N2 strain with Sb1079, Sc16, Dh25, and ST, instead of *E. coli* OP50 (normally used as food to maintain the nematode), affected the longevity of the *C. elegans* in a solid medium (Figure 3a). Sb1079 and ST reduced the mean lifespan by 2 days compared to the worms fed on *E. coli* OP50 (9 days versus 11 days), whereas Sc16 and Dh25 increased it by 1 day and 4 days, respectively. On the other hand, the three tested yeasts increased longevity from 4 to 7 days, in comparison with *E. coli* OP50. The two cheese yeasts had a beneficial effect on *C. elegans* wild-type (*p* < 0.001), whereas ST presented a low pathogenicity (*p* < 0.001). Recently, Kunyeit et al. [12] showed that *Saccharomyces cerevisiae* (strain KTP) and *Issatchenkia occidentalis* (strain ApC), had a negative effect on the mean lifespan. These results are not in accordance with our data, suggesting a yeast-strain-dependent effect on *C. elegans*.

In a liquid medium (Figure 3b), the mean lifespans of the worm strain N2 fed on *E. coli* OP50 and ST were equal to 7 and 8 days, respectively. The mean lifespan was equal to 12 days with Sb1079 and Dh25, while increasing to 17 days for Sc16. The three yeasts increased survival (19, 30, and 31 days for Sb1079, Dh25, and Sc16, respectively) in comparison with OP50 and ST (13 and 15 days, respectively). The variation in the lifespan of *C. elegans*, between cheese yeasts and E. coli OP50, might be explained by the different sources of nutritional components. Thus, it could be interesting to measure the body sizes, the lipid accumulation, and the fertility of *C. elegans* in line with the different nutrition sources. In our conditions, the three tested yeasts showed a significant pro-survival effect on *C. elegans* (*p* < 0.001). The *C. elegans* N2 lifespan was not significantly (*p* = 0.313) different between the ST and *E. coli* OP50 conditions. The serovar Typhimurium could be virulent in *C. elegans* [64,65], but in our in vivo experimental conditions, the *Salmonella* cheese strain may be considered avirulent.

We then used the worm strain AU37 for its high sensitivity to pathogens. When this *C. elegans* was fed on ST, the mean lifespan decreased to one day, compared to 7 days with *E. coli* OP50 (Figure 3c). This result showed the high virulence of ST on this mutant worm strain. Dh25 increased (*p* < 0.001) mean lifespan and longevity significantly, confirming the beneficial effects of this cheese yeast. When *E. coli* OP50 and Dh25 were tested as a preventive treatment against ST, the lifespan increased from 1 day with ST alone, to 3 and 4 days with Dh25 and *E. coli* OP50, respectively. It could be interesting to further investigate whether the protection procured by *E. coli* OP50 and Dh25 arose from the inhibition of the intestinal colonization of *S.* Typhimurium. Zhou et al. [66] showed that the action of *Lactobacillus* against Enterotoxigenic *Escherichia coli* in *C. elegans* was not due to colonization inhibition but to the suppression of pathogen enterotoxin gene expression. To further characterize the molecular mechanism involved in the observed increase in lifespan, we could follow the nuclear translocation of the DAF-16 transcription factor using the transgenic TJ-356 worm strain. Indeed, Tullet et al. [67] reported the importance of DAF-16 in the extension of lifespan through the insulin/IGF-1 metabolic signaling pathway. In addition, the DAF-16 implication has been demonstrated by Poupet et al. [39] in the preventive effects of a probiotic bacterium against a pathogenic yeast, *Candida albicans*.

## 4. Conclusions

In the present study, we investigated the relevance of in vitro and in vivo methods in the screening of cheese yeasts as probiotic candidates in addition to the well-known *S. cerevisiae* and *S. boulardii*. Our data showed that the yeast *Debaryomyces hansenii* 25, isolated from cheese, exhibits interesting probiotic traits. This strain shows a clear resistance to low pH (pH 2.5) and to bile contact. It also expresses a capacity to adhere to Caco-2 and HT29-MTX intestinal epithelial cells and leads to induced TEER enhancement similar to the probiotic *Saccharomyces cerevisiae* subspecies *boulardii*. Furthermore, the lengthening of the co-culture duration between *D. hansenii* 25 and *S.* Typhimurium UPsm1 delays *Salmonella*-induced TEER disruption and could protect human epithelial cells from pathogen invasion. This result is further confirmed by the protective effects of the yeast against *S.* Typhimurium UPsm1 in *C. elegans*. We also highlight the importance of the choice of the nematode strain according to the virulence of the pathogen tested. Thus, the combination of in vitro and in vivo methods, before human clinical trials, like the measurement of epithelial cell barrier function and *C. elegans* lifespan assay, can improve efficiency when selecting probiotic yeast candidates. The nematode can be used as a preclinical model to study the preventive effect of probiotic yeasts against pathogens. Additional investigations to decipher the mechanisms involved in the resistance provided by *D. hansenii* 25 against *S.* Typhimurium UPsm1 in *C. elegans* are however needed. Transcriptomic approaches and/or the use of loss-of-function mutant worms targeting signal transduction pathways implicated in immunity could be of great interest.

The fact that the strain *D. hansenii* 25 has probiotic characteristics and that the other cheese yeasts tested resist the conditions simulating passage through the gastrointestinal tract suggests that the consumption of cheeses could have a favorable impact on human health via the activity of yeasts.

## Figures and Tables

**Table 1:** *D. hansenii* 25 and *S. cerevisiae* 16 in the presence of acid pH (2.5). (**a**) Acid pH tolerance (%) = (number of colony-forming units (CFU) in broth with pH = 2.5/number of CFUs in broth with pH = 7) X 100. (**b**) The survival rate of *S. cerevisiae* subspecies *boulardii* CNCM I/079, *D. hansenii* 25 and *S. cerevisiae* 16 in the presence of bile salts (3%). Bile tolerance (%) = (number of CFUs in broth with bile/number of CFUs in broth without bile) X 100. The values are mean ± SD (error bars) of three independent experiments. Results show a high resistance to acidic pH and bile salts, suggesting the strains will be able to resist to gastric environment.

**a.** Survival rates in the presence of acidic pH (%)				
**Strains**	**45 min**	**90 min**		
*S. boulardii* 1079	93.6 ± 8.3	91.1 ± 9.0		
*D. hansenii* 25	96.6 ± 3.4	85.5 ± 3.0		
*S. cerevisiae* 16	101.3 ± 3.4	54.2 ± 2.7		
**b.** Survival rates in the presence of bile salts (%)				
**Strains**	**1 h**	**2 h**	**3 h**	**4 h**
*S. boulardii* 1079	85.3 ± 4.5	70.3 ± 4.4	63.1 ± 2.7	73.5 ± 4.9
*D. hansenii* 25	94.1 ± 7.4	91.3 ± 7.7	72.8 ± 13.5	68.9 ± 16.1
*S. cerevisiae* 16	89.2 ± 2.9	77.5 ± 15.1	56.0 ± 9.4	39.2 ± 6.7

**Table 2:** Determination of the number of viable adhering *S.* *boulardii* 1079, *D. hansenii* 25, and *S. cerevisiae* 16 strains after 1 h of contact with (**a**) HT29-MTX and with (**b**) Caco-2, cell lines at multiplicities of infection (MOI) of 0.1, 1, 10, and 100. Results are expressed as percentages of adhering yeasts compared to the initial inoculum (mean ± SD). a, b superscripts represent groups determined by Least Significant Difference (LSD) test, the same letter indicating values not significantly different (*p* < 0.05 by ANOVA1) across a column. Results indicate yeast adhesion is higher on Caco-2 cells than on HT29-MTX cells.

**a.** Multiplicity of infection (MOI)				
**HT29-MTX**	**MOI 100**	**MOI 10**	**MOI 1**	**MOI 0.1**
*S. boulardii* 1079	0.51 ± 0.046% ^a,b^	0.39 ± 0.123% ^a,b^	0.46 ± 0.189% ^a,b^	1.15 ± 0.270% ^a,b^
*D. hansenii* 25	0.18 ± 0.005% ^b^	0.29 ± 0.005% ^b^	0.44 ± 0.007% ^b^	1.56 ± 0.010% ^b^
*S. cerevisiae* 16	0.91 ± 0.015% ^a^	0.90 ± 0.013% ^a^	1.24 ± 0.020% ^a^	2.61 ± 0.024% ^a^
**b.** Multiplicity of infection (MOI)				
**Caco-2**	**MOI 100**	**MOI 10**	**MOI 1**	**MOI 0.1**
*S. boulardii* 1079	0.59 ± 0.006% ^a^	1.87 ± 0.011% ^a^	1.77 ± 0.013% ^a,b^	3.60 ± 0.028% ^a^
*D. hansenii* 25	2.27 ± 0.011% ^b^	2.50 ± 0.060% ^a^	4.10 ± 0.325% ^b^	4.12 ± 1.701% ^a^
*S. cerevisiae* 16	0.81 ± 0.009% ^a^	0.86 ± 0.007% ^b^	2.56 ± 0.032% ^a^	7.26 ± 0.088% ^a^

**Table 3:** Effect of the control yeast strain, *S. cerevisiae* subspecies *boulardii* CNCM I-1079 (Sb1079), *D. hansenii* 25 (Dh25) and *S. cerevisiae* 16 (Sc16), isolated from cheese, on *S.* Typhimurium (ST) growth. a, b superscripts represent groups determined by LSD test, the same letter indicating values not significantly different (*p* <0.05 by ANOVA1) across a column. The yeasts tested have an inhibition activity against *Salmonella* growth.

Time (h)	0	24	48
ST	6.47 ± 0.48 ^a^	8.88 ± 0.30 ^a^	8.71 ± 0.20 ^a^
ST + Sb1079	6.63 ± 0.38 ^a^	8.39 ± 0.50 ^a,b^	6.46 ± 0.92 ^b^
ST + Dh25	6.58 ± 0.46 ^a^	8.34 ± 0.62 ^b^	6.90 ± 0.97 ^b^
ST + Sc16	6.60 ± 0.43 ^a^	8.21 ± 0.43 ^b^	6.91 ± 0.97 ^b^

**Table 4:** Effect of *D. hansenii* 25 (Dh25), isolated from cheese, on *S.* Typhimurium (ST) growth after four co-culture durations (24 h, 48 h, 72 h, and 96 h). a, b, and c superscripts represent groups determined by LSD test, a same letter indicating values not significantly different (*p* < 0.05 by ANOVA1) with t = 0 h as the control.

Co-Culture Duration	ST (log CFU/mL)	Dh25 (log CFU/mL)
0 h	6.40 ± 0.10 ^a^	5.93 ± 0.21 ^a^
24 h	8.08 ± 0.58 ^b^	6.60 ± 0.35 ^a^
48 h	6.90 ± 0.13 ^a^	5.90 ± 0.56 ^a^
72 h	6.85 ± 0.30 ^a^	4.87 ± 0.81 ^b^
96 h	6.79 ± 0.09 ^a^	4.15 ± 0.85 ^c^

## Supplementary Materials

The following are available online at https://www.mdpi.com/2076-2607/8/6/922/s1. Table S1: Survival rates of yeasts strains in the presence of acid pH (2.5). a, acid pH tolerance (%) = number of CFUs in broth with pH = 2.5/number of CFUs in broth with pH = 7) X 100. b, the survival rate of *S. cerevisiae* subspecies *boulardii* CNCM I/079, *D. hansenii* 25 and *S. cerevisiae* 16 in the presence of bile salts (3%). Bile tolerance (%) = number of CFUs in broth with bile/number of CFUs in broth without bile) X 100. The values are mean ± SD (error bars) of three independent experiments. Results show a high resistance to acidic pH and bile salts suggesting the strains will be able to resist to gastric environment. Figure S1: Evolution of Caco-2 cells TEER during maturation of the cell layer.

## Abbreviation

TEER	TransEpithelial Electrical Resistance
