# Supplementary material for: Investigation into In Vitro and In Vivo Caenorhabditis elegans Models to Select Cheese Yeasts as Probiotic Candidates for their Preventive Effects against Salmonella Typhimurium
Source: Microorganisms. 2020 Jun 18;8(6):922. doi: 10.3390/microorganisms8060922 (PMC7356738; doi:10.3390/microorganisms8060922)

Table S1: Survival rates of yeasts strains in the presence of acid pH (2.5). **a,** acid pH tolerance (%) = number of CFUs in broth with pH = 2.5/number of CFUs in broth with pH = 7) X 100. **b,** the survival rate of *S. cerevisiae* subspecies *boulardii* CNCM I/079, *D. hansenii* 25 and *S. cerevisiae* 16 in the presence of bile salts (3%). Bile tolerance (%) = number of CFUs in broth with bile/number of CFUs in broth without bile) X 100. The values are mean ± SD (error bars) of three independent experiments. Results show a high resistance to acidic pH and bile salts suggesting the strains will be able to resist to gastric environment.

a. Survival rates in the presence of acidic pH (%)

**Strains**	**45 min**	**90 min**
K. lactis 1	84.1 ± 3.7	55.6 ± 3.2
K. lactis 6	48 ± 1.8	48 ± 1.8
D. hansenii 14	81.4 ± 2.9	60.2 ± 3.2
D. hansenii 25	96.6 ± 3.4	85.5 ± 3.0
Y. lipolytica 4PO1	83.3 ± 4.1	61.1 ± 2.9
Y. lipolytica 3CP5	80.0 ± 2.6	49 ± 2.5
S. boulardii 1079	93.6 ± 8.3	91.1 ± 9.0
S. cerevisiae 52	62.9 ± 3.7	60.0 ± 3.1
S. cerevisiae 16	101.3 ± 3.4	54.2 ± 2.7

b. Survival rates in the presence of bile salts (%)

**Strains**	**1 h**	**2 h**	**3 h**	**4 h**
K. lactis 1	77 ± 3.3	196 ± 15.1	61 ± 1.9	148 ± 9.9
K. lactis 6	82 ± 3.5	49 ± 4.3	29 ± 3.2	36 ± 3.3
D. hansenii 14	102 ± 8.1	85 ± 3.7	78 ± 2.5	68 ± 12.4
D. hansenii 25	94.1 ± 7.4	91.3 ± 7.7	72.8 ± 13.5	68.9 ± 16.1
Y. lipolytica 4PO1	102 ± 7.6	85 ± 6.1	78 ± 1.8	68 ± 6.4
Y. lipolytica 3CP5	131 ± 8.9	95 ± 5.3	174 ± 7.7	262 ± 7.7
S. boulardii 1079	85.3 ± 4.5	70.3 ± 4.4	63.1 ± 2.7	73.5 ± 4.9
S. cerevisiae 52	67 ± 13.2	59 ± 2.6	45 ± 2.5	65 ± 2.3
S. cerevisiae 16	89.2 ± 2.9	77.5 ± 15.1	56.0 ± 9.4	39.2 ± 6.7

Figure S1: Evolution of Caco-2 cells TEER during maturation of the cell layer.

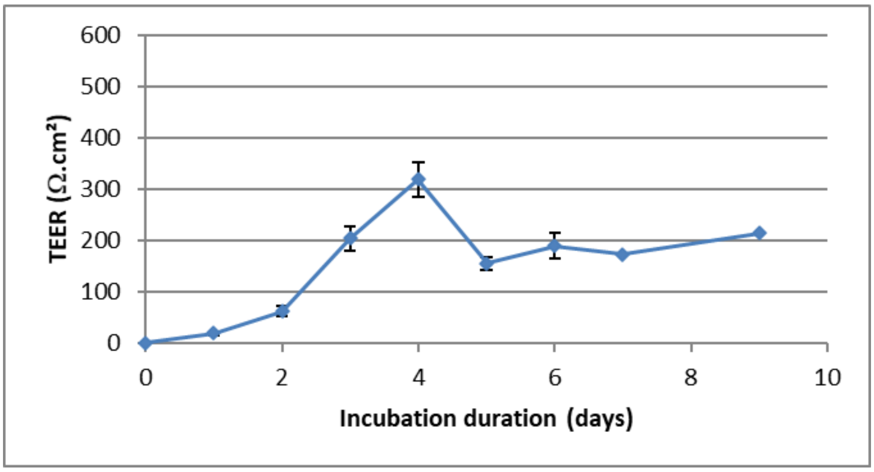

Supplement: Supplementary file 1 [file microorganisms-08-00922-s001.pdf]
